# Seizures Induced by Pentylenetetrazole in the Adult Zebrafish: A Detailed Behavioral Characterization

**DOI:** 10.1371/journal.pone.0054515

**Published:** 2013-01-21

**Authors:** Ben Hur M. Mussulini, Carlos E. Leite, Kamila C. Zenki, Luana Moro, Suelen Baggio, Eduardo P. Rico, Denis B. Rosemberg, Renato D. Dias, Tadeu M. Souza, Maria E. Calcagnotto, Maria M. Campos, Ana M. Battastini, Diogo L. de Oliveira

**Affiliations:** 1 Departamento de Bioquímica, Universidade Federal do Rio Grande do Sul, Porto Alegre, Rio Grande do Sul, Brazil; 2 Instituto de Toxicologia e Farmacologia, Pontifícia Universidade Católica do Rio Grande do Sul, Porto Alegre, Rio Grande do Sul, Brazil; VIB & Katholieke Universiteit Leuven, Belgium

## Abstract

Pentylenetetrazole (PTZ) is a common convulsant agent used in animal models to investigate the mechanisms of seizures. Although adult zebrafish have been recently used to study epileptic seizures, a thorough characterization of the PTZ-induced seizures in this animal model is missing. The goal of this study was to perform a detailed temporal behavior profile characterization of PTZ-induced seizure in adult zebrafish. The behavioral profile during 20 min of PTZ immersion (5, 7.5, 10, and 15 mM) was characterized by stages defined as scores: (0) short swim, (1) increased swimming activity and high frequency of opercular movement, (2) erratic movements, (3) circular movements, (4) clonic seizure-like behavior, (5) fall to the bottom of the tank and tonic seizure-like behavior, (6) death. Animals exposed to distinct PTZ concentrations presented different seizure profiles, intensities and latencies to reach all scores. Only animals immersed into 15 mM PTZ showed an increased time to return to the normal behavior (score 0), after exposure. Total mortality rate at 10 and 15 mM were 33% and 50%, respectively. Considering all behavioral parameters, 5, 7.5, 10, and 15 mM PTZ, induced seizures with low, intermediate, and high severity, respectively. Pretreatment with diazepam (DZP) significantly attenuated seizure severity. Finally, the brain PTZ levels in adult zebrafish immersed into the chemoconvulsant solution at 5 and 10 mM were comparable to those described for the rodent model, with a peak after a 20-min of exposure. The PTZ brain levels observed after 2.5-min PTZ exposure and after 60-min removal from exposure were similar. Altogether, our results showed a detailed temporal behavioral characterization of a PTZ epileptic seizure model in adult zebrafish. These behavioral analyses and the simple method for PTZ quantification could be considered as important tools for future investigations and translational research.

## Introduction

Epilepsy is a neurological disorder characterized by recurrent spontaneous epileptic seizures associated with distinct neurobiological and behavioral alterations [1]. One of the methods used to investigate epileptic seizures in experimental models consists on the analysis of the behavioral profile through a seizure stage-score classification [2]. This characterization is well established in rodents for seizures induced by electrical kindling Racine et al. [3] and for chemoconvulsant drugs, such as kainate (KA), pilocarpine and pentylenetetrazole (PTZ) [4]. Exposure to PTZ induces a concentration-dependent sequence of stereotyped behavioral changes that starts with orofacial movements and culminates in clonus-like seizures in rodent models. This seizure model has been widely used in the past 6 decades for discovery and development of several antiepileptic drugs (AED), such as benzodiazepines, valproate, gabapentine, etc [5], [6], [7]. Despite the advances in new AED discovery, 30% of epileptic patients still suffer with refractory epilepsy [8], [9]. Löscher et al. [5], in a critical review about the current animal models of seizure and epilepsy employed to discovery and development of new AED, pointed out that this high refractoriness could be a result from using always the same pro-convulsant focusing in rodent models.

In this context, the zebrafish (*Danio rerio*) emerges as a new animal model to evaluate the effects of classical pro-convulsant drugs in order to develop and characterize new AED [10]–[14]. This species exhibits several anatomic similarities and a high genetic homology with mice and humans [15], [16]. Moreover, zebrafish presents a tight junction-based blood–brain barrier similar to higher vertebrates, with substantial macromolecule permeability, which makes this model an attractive organism for high throughput screening applications and AED discovery [17], [18]. Furthermore, this model offers a potential non-discriminatory screening for AED [13], in contrast to that for rodents where usually seizures induced by PTZ were used to identify anticonvulsants agents that acts mainly through GABA [19]. All these aspects contributes to an increased number of investigations involving chemoconvulsant induced-seizures in zebrafish, and epilepsy research through genetic models that are either susceptible or resistant to seizures, and mutations associated with known human epilepsy syndromes [20]–[29]. These investigations are based mostly in Baraban et al. [20] study, which showed that zebrafish larvae exposed to PTZ displays complex and stereotypical patterns of seizure behavior sequence, ictal- and interictal-like electrical activity in immobilized animal, and *c-fos* expression in brain regions. However, the use of adult zebrafish, which shows a broader behavioral repertoire [30]–[33], with a fully developed central nervous system (CNS), when compared to zebrafish larvae [34], [35], could improve the currently protocols for new AED research and to study the mechanisms underlying seizures.

Although electrophysiology pattern of PTZ induced seizure activity [31], *c-fos* expression [32], and some behavioral analyses have already been demonstrated in adult zebrafish exposed to PTZ [22], [36], [37], questions regarding the behavioral profile of the seizure pattern remain unanswered, such as: (I) Is the score system proposed by Desmond et al. [35] for studying seizure in adult zebrafish suitable for all behavior seizure manifestations in PTZ model?; (II) What is the sequence of behaviors during the exposure to PTZ?; (III) Are these manifestations similar to larvae?; (IV) Does the seizure behavior profile change with alterations of drug concentration?; (V) What is the latency to reach the clonus-like behavior in distinct concentrations of PTZ exposure?; (VI) How long does animals take to return to a normal behavior after PTZ exposure?; (VII) Does the PTZ levels in the fish brain depends on the concentration and time of exposure?; (VIII) What is the mortality rate of fish exposed to PTZ at distinct concentrations?

In order to answer these questions, we performed a detailed behavioral seizure analysis of PTZ-induced seizures in adult zebrafish. Additionally, we performed a quantification of brain PTZ levels during and after exposure to this chemoconvulsant drug. All of these analysis were based on the pioneer work performed by Alfaro et al. [21] where they characterized a detailed seizure scale for kainic acid-induced epileptic seizures in adult zebrafish.

## Materials and Methods

### Ethics Statement

All procedures with animal subjects have been approved by the Ethic Committee for Use of Animals - CEUA from Universidade Federal do Rio Grande do Sul (protocol number 22214).

### Reagents

Pentylenetetrazole (PTZ) was purchased from Sigma-Aldrich (St. Louis, MO, USA). Diazepam (DZP) was purchased from União Química Nacional S/A (Pouso Alegre, MG, Brazil). Acetonitrile and methanol were purchased from Merck® (Darmstadt, Hessen, Germany). Pure Ultra pure water was obtained from a Millipore Corporate® Milli-Q water system (Billerica, Massachusetts, USA). All HPLC components and software ChemStation were from Agilent Technologies® Inc. Santa Clara, California, USA.

### Animals

Adult zebrafish (*Danio rerio*; 4 to 6 months-old, ±50:50 male:female ratio) of heterogeneous wild-type stock (standard short-fin phenotype) were obtained from a local commercial supplier (Delphis, RS, Brazil). Animals were carefully weighted and measured in order to select the ones with similar weight and size (35±2 mg and 2±0.15 cm, respectively) to avoid putative variations of drugs pharmacodynamic and pharmacokinetic. Fish were housed in 50-L aquariums (80-100 fish per aquarium) for at least 2 weeks prior to the experiments in order to acclimatize to the animal facility. All tanks were filled with non-chlorinated water previously treated with 132 µL/L AquaSafe® (Tetra, VA, USA) and kept under mechanical and chemical filtration at a targeted temperature of 26±2°C and water pH at 7.0 to 8.0 (system water). The room illumination was provided by ceiling-mounted fluorescent lamps on a 14/10 light/dark photo period cycle (lights on at 7:00 am). Animals were fed twice a day with a commercial flake fish food (Alcon BASIC®, Alcon, Brazil). All animals used in this study were experimentally naive, healthy and free of any signs of disease. They were maintained according to the National Institute of Health Guide for Care and Use of Laboratory Animals (2011).

### Treatments and Seizure Behavioral Characterization

To induce experimental epileptic seizures, animals (*n* = 12 in each group) were individually exposed to 5, 7.5, 10, and 15 mM PTZ, readily dissolved in water. All PTZ concentrations and the time of exposure were based on previous reports in order to induce clonus-like seizure responses [20], [22], [36], [38], [39]. The control group was exposed to system water only. In order to investigate the effect of a classical AED on PTZ-induced seizures in zebrafish, a GABA_A_ positive allosteric modulator diazepam (DZP) was used. Two groups of animals were exposed to 75 µM DZP for 40 min in a beaker containing a 0.5-L solution. Afterwards, the animals were rapidly transferred to a beaker containing system water to remove the excess of DZP. One group was further transfer to a tank with system water to investigate the DZP sedative effect (DZP control group) and another group to a similar tank containing 10 mM PTZ solution (DZP/10 mM PTZ group). All conditions for DZP experiments were previously set up by our group (data not shown).

The detailed behavioral seizure profile characterization was performed during the same time frame each day (from 10:00 am to 4:00 pm). The apparatus consisted of a tank (20 cm width×13 cm height×7 cm length) filled with 1.5 L of PTZ solution or system water. In order to keep the same experimental conditions, animals were randomly handled from their home tanks and individually transferred to beakers filled only with system water for the same period of DZP pretreatment (40 min). Animals were carefully placed individually in the tanks and their behavioral seizure activity was recorded for a single session of 20 min. At each experiment a fish was placed individually into the determined treatment solution, which was not used in subsequent experiments. Assays were performed at three independent days with 4 animals in each group per day. All experimental procedures were performed on a silent room.

A webcam (Microsoft® LifeCam 1.1 with Auto-Focus) was placed 30 cm from the testing tank to ensure good video recording and to monitor the location and swimming activity of the fish. All tank walls were coated with white background cover, in order to avoid the reflex of the animal in the walls and in the tank bottom, and to ensure a uniform background for the video analysis. To boost the contrast between the background and zebrafish, two 60-watts light bulbs were placed 50 cm behind the tanks. All behavioral data were evaluated by two trained observers in a blinded fashion (inter-rater reliability ≥0.92). All necessary precautions were taken to ensure representative behavioral results and also to avoid handling stress. Throughout the experiments, the fish were gently transferred between home tanks, beakers, and experimental apparatus. All fish were handled and tested at similar way and the behaviors were recorded in the same room, which kept the manipulation, water quality, and illumination uniform and constant between trials.

### Epileptic Seizure Stage Score

The fish (*n* = 6) were immersed into a PTZ solution (5–15 mM) and monitored for 60 min to evaluate epileptic seizures-related behavior. To characterize each stage, behavioral manifestations were evaluated according to the literature [22], [35], [37] and the sequence of behavioral manifestations was described using a range from lower to higher concentration of PTZ tested in our study. It is important to emphasize that random alterations in behavioral induced by PTZ (e.g., jumping) were not considered. For each stage, we assigned a specific score, described in Table 1 (see Video S1).

**Table 1:** Score phenotype of the PTZ seizure model in adult zebrafish.

SCORE	Behavior phenotype
0	Short swim mainly in the bottom of the tank.
1	Increased swimming activity and high frequency of opercular movement.
2	Burst swimming, left and right movements, and erratic movements.
3	Circular movements.
4	Clonic seizure-like behavior (abnormal whole-body rhythmic
	muscular contraction).
5	Fall to the bottom of the tank, tonic seizure-like behavior (sinking to the bottom of
	the tank, loss of body posture, and principally by rigid extension of the body).
6	Death.

All behavior phenotypes present in video S1 defined by scores.

### Washout Period and Survival Assessments

**Figure 1:**
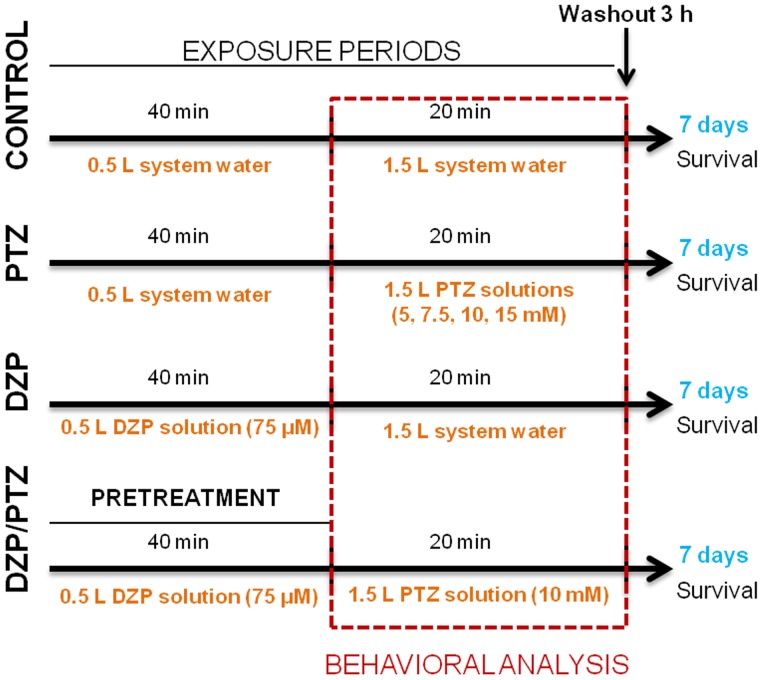
Experimental protocol schematic representation. The methodological approach used to evaluate the epileptic seizure-like behavior (experimental conditions, exposure periods, washout period, and survival evaluation after behavioral tests).

After PTZ exposure, we transferred the fish to an intermediary beaker containing system water to eliminate all the residual PTZ in contact with the animal. Immediately after, we than transferred the fish to another beaker with a new clean system water to evaluate the washout period. During the washout of PTZ, groups were observed for more three hours, to determine the latency to return to score 0. We considered that the animal fully returned to the basal behavior when it reached the score 0 and remained in this score until the end of the 3 hours. In order to access the survival rate, the fish were individually transferred to 1 L recipient, filled with system water. The water was renewed every 24 h and the survival was assessed (the total time of this protocol was 168 h). The Figure 1 illustrates the protocol used in this study.

### Determination of PTZ Levels in the Zebrafish Brain

To quantify the PTZ levels that reached the zebrafish brain, we performed experiments using high-performance liquid chromatography equipped with an isocratic pump, diode array detector (DAD), degasser and manual injection system. Chromatographic separations were performed using a reverse-phase column (250 mm×4 mm, 5 mm LiChrospher® 100 RP-18). The column was protected by a guard column (4×4 mm, 5 mm LiChrospher® 100 RP-18) and maintained at a temperature 22±2°C. The mobile phase was a mixture of phosphate buffer 10 mM pH 6.9: methanol: acetonitrile (60:35:5, v/v/v). The flow rate of 0.8 mL/min was maintained isocratically, the DAD was set at 202 nm and the total run time was 6 minutes.

The PTZ determination was based on the method previously described by Soto-Otero et al. [40]. Each independent experiment was performed using a pool of three whole brains of animals from the PTZ groups (5 mM and 10 mM) after the exposure of 2.5 min, 20 min, and after 60 min PTZ washout. Briefly, the animals underwent cryoanesthesia and euthanized by decapitation. The cranial skulls were excised; the brains were removed and rapidly homogenized in 1 mL of cold PBS using a glass–Teflon homogenizer in ice. The samples (*n* = 4 per group) were centrifuged at 13.500 *g* for 5 min at 4°C in 1.5 mL tubes, and the supernatants were collected for PTZ analysis. Nine hundred microliters of supernatant was treated with 5.0 mL of dichloromethane. The tube was mixed for 30 s, an excess of ammonium sulfate was added with calibrated spatula and the tube contents were mixed again for 30 s. The samples were centrifuged at 800 *g,* the organic phase was separated and totally dried with nitrogen at room temperature. Following, the dried samples were reconstituted with 100 μL of the mobile phase and 20 μL was injected into HPLC. Standard curve measures (1–75 µg/mL) can be accessed in Figure S1.

### Statistics

Non-parametric data of seizure scores were expressed as median ± interquartile range. Scatter plots were designed to enable the analysis of variance across time performed by Friedman test followed by Dunn’s Multiple Comparison test as post hoc. Cumulative frequency was determined using the percentage of animal that reached each score across time for the respective treatment tested. The area under the curve (AUC), latency, and washout period were represented as mean ± S.E.M and analyzed by the one-way ANOVA followed by the Bonferroni’s test as post hoc. Student’s *t* test was used to compare the DZP/10 mM PTZ and 10 mM PTZ groups. The PTZ quantification in brain were expressed as mean ± S.E.M and analyzed by two-way ANOVA followed by Bonferroni’s test post-hoc. The survival time was compared among groups using the log-rank test of trend. In all analyses, the significance level was taken as *p*≤0.05.

## Results

**Figure 2:**
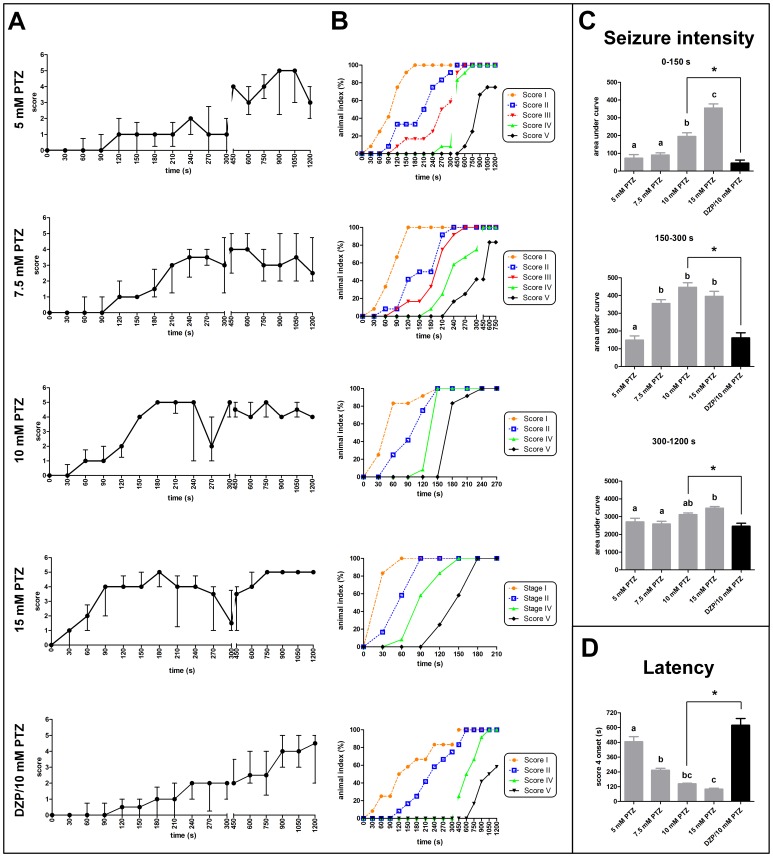
Behavioral profile of PTZ-induced seizures in adult zebrafish. The main characteristic seizure behavior induced by 5–15 mM PTZ and DZP/10 mM PTZ treatments during 20 min (*n* = 12). (A) Seizure score (only the highest score reached was consider in each interval) and cumulative frequency (B). Data are represented as median ± interquartile range and as the animal index (%) that reached the scores across time, respectively. (C) Seizure intensity during distinct moment tests (0–150, 150–300, and 300–1200 s) evaluated by the area under curve observed for each treatment. (D) Latency to score 4 onset. Data from seizure intensity and latency are represented as mean ± S.E.M and analyzed by one-way ANOVA followed by Bonferroni’s test as post-hoc. Distinct letters indicate statistical differences among PTZ-treated groups (gray bars). The DZP/10 mM PTZ is represented as black bars and compared to 10 mM PTZ group by the Student’s *t* test. *indicates significant difference between groups.

The behavioral analysis shown that the control group exhibited spontaneous usual swimming movements consisted by repeated short swims (data not shown). On the other hand, animals immersed into PTZ solution presented behavioral epileptic seizures, classified in different scores as shown in Table 1. Figure 2A depicts the temporal behavioral profile of animals exposed to different PTZ concentrations. There was a rapid score progression in the first 5-min period for 10 and 15 mM PTZ (Friedman test, *p*<0.0001; Dunn’s Multiple Comparison test, *p*<0.05). Therefore, in order to perform an analysis across time, the first 5 min and the remaining 15 min were divided into 30 s and 150 s intervals, respectively. Animals immersed into the higher concentrations (10 and 15 mM) reached scores 4 and 5 faster than animals exposed to lower concentrations (5 and 7.5 mM) of PTZ. Animals immersed into 5 mM PTZ showed repetitive low scores (1 and 2) during the first 5-min and only presented scores 4 and 5 in the last 15-min period. Animals exposed to 7.5 mM PTZ presented an intermediate profile between 5 mM and higher concentrations, alternating from scores 3 to 5, starting at 210 to 1200 s (Friedman test, *p*<0.0001; Dunn’s Multiple Comparison test, *p*<0.05).

The time necessary to reach score 5 were: 1050 s for 5 mM; 600 s for 7.5 mM; 240 s for 10 mM; and 180 s for 15 mM. Furthermore, the animals immersed into 10 and 15 mM PTZ showed a rapid change from scores 0–2 to score 4 (16.66 and 58.33%, respectively) (Figure 2B). The score 5 was not observed in all animals treated with lower concentrations (only 83.33% and 75% of animals reached this score at 7.5 mM and 5 mM, respectively). Scatter plot representations were performed in order to see each animal behavioral seizure profile across time described in the score curves (Figure S2).

The analysis of the score curves (Figure 2A) suggests that there are three different moments for the PTZ-induced seizures in zebrafish. In the first moment (0 to 150 s), higher PTZ concentrations induced seizures with score 4; in the second (150 to 300 s), PTZ at 7.5 mM induced seizures with scores 3–4; and in the third (300 to1200 s), all PTZ concentrations induced seizures with scores 3–5 (Figure S3A). In order to evaluate the seizure intensity across time in these three moments, we measured the area under score curve for each animal for each PTZ concentration (Figure 2C). In the first interval (0–150 s), animals from 15 mM PTZ group presented higher seizure intensity than 10 mM PTZ and both concentrations displayed higher seizure intensity when compared to 5 and 7.5 mM (one-way ANOVA, *F* [4,59] = 44.56, *p*<0.0001; Bonferroni test, *p*<0.05). In the second interval (150–300 s), the seizure intensity was lower in 5 mM PTZ group when compared with other groups (one-way ANOVA, *F* [4,59] = 28.12, *p*<0.0001; Bonferroni test *p*<0.05). In the last interval (300–1200 s), animals from 15 mM PTZ group showed a higher seizure intensity when compared to 5 and 7.5 mM PTZ (one-way ANOVA, *F* [4,59] = 20.10, *p*<0.0001; Bonferroni test, *p*<0.05). Figure S3B shows the seizure intensity during the total time of observation (1200 s).

**Figure 3:**
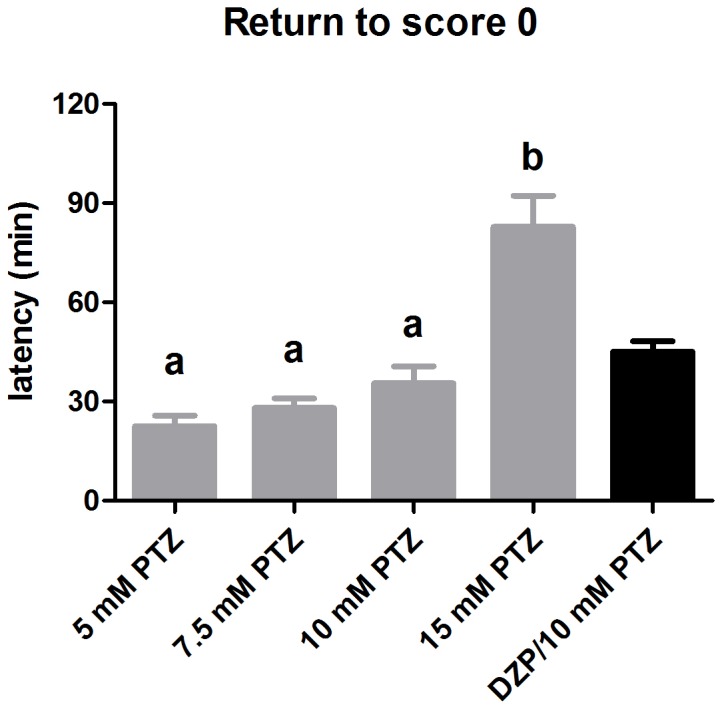
Latency to score 0 during the washout period. Data are represented as mean ± S.E.M and analyzed by one-way ANOVA followed by Bonferroni’s test as post-hoc. Distinct letters indicate statistical differences among PTZ-treated groups (gray bars). The DZP/10 mM PTZ is represented as black bars and compared to 10 mM PTZ group by Student’s *t* test with no statistical difference.

Animals immersed into 15 mM PTZ solution showed lower latency to the first episode of seizure score 4 when compared to animals immersed into 5 and 7.5 mM PTZ. In addition, animals exposed to 5 mM PTZ presented higher latency to the score 4 when compared to all other concentrations (one-way ANOVA, *F* [4,59] = 49.43, *p*<0.0001; Bonferroni test, *p*<0.05), (Figure 2D). In the washout period only 15 mM PTZ group required a longer time to return to score 0, when compared to other concentration groups (one-way ANOVA; *F* [4,59] = 21.16, *p*<0.0001; post-hoc, *p*<0.05) (Figure 3).

**Figure 4:**
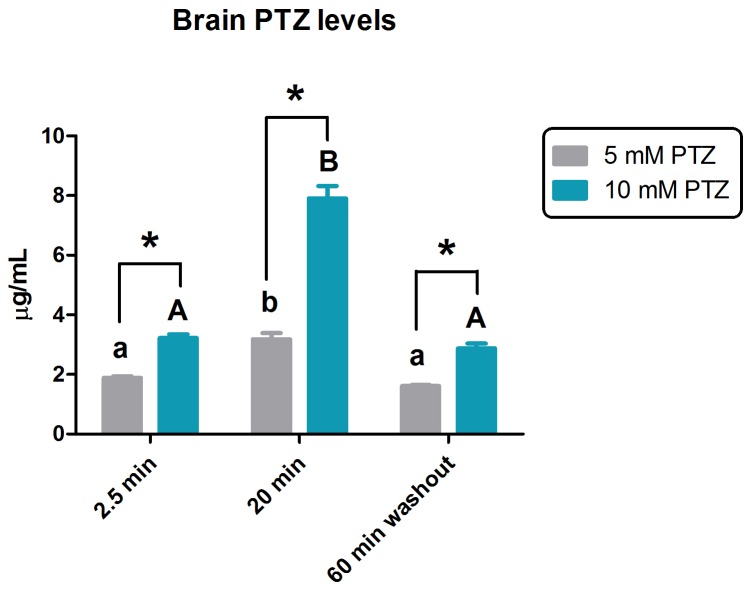
Quantification of the PTZ levels in the brain. The concentration of PTZ in the brain was determined as μg PTZ/mL sample at 5 mM (gray bars) with values of 1.885, 3.175, 1.612, and 10 mM (blue bars) with values of 3.222, 7.905, and 2.870, after 2.5 and 20-min of PTZ exposure, and after 60-min washout. Data are expressed as mean ± S.E.M and analyzed by two-way ANOVA followed by Bonferroni’s test as post-hoc. Distinct letters indicate statistical difference within the same group at different periods, (lower case letter for 5 mM and capital letters for 10 mM) whereas the asterisks (*) indicates significant difference between both PTZ groups for each time.

As shown in Figure 4, higher PTZ levels in the brain were found in animals exposed to 10 mM PTZ when compared with 5 mM PTZ (two-way ANOVA, concentration effect, *F* [1], [12] = 152.4, *p*<0.0001). Additionally, there was a peak of PTZ in the brain after 20-min of exposure. Similar levels were observed after a 2.5-min exposure and after a 60-min removal from the PTZ solutions (two-way ANOVA, time effect, F [2], 12 = 176.9, *p*<0.0001). There was a positive interaction between exposure time and PTZ concentration (two-way ANOVA, time X concentration effect: *F* [2], [12] = 52.41, *p*<0.0001; Bonferroni test, *p*<0,01).

After the DZP pretreatment, fish remained immobilized during the initial 7 min of observation. Afterwards, fish began to present short movements in the bottom of the tank and, after 15 min the motor activity was recovered, being similar to the control group. Two out of 12 animals immersed into DZP died during observation (data not shown). Zebrafish from DZP/10 mM PTZ group presented different temporal seizure profile compared with 10 mM PTZ group (Figure 2A). In 5 min, all animals exposed to 10 mM PTZ reached scores 4 and 5. However, 66% of animals exposed to DZP/10 mM PTZ presented scores 1 and 2, 16% reached score 3 and 16% exhibited score 0. In the last 15 min, animals exposed to 10 mM PTZ presented an alternation between scores 4–5. On the other hand, fish pretreated with DZP presented scores from 2 to 5 (Friedman test, *p*<0.05; Dunn’s Multiple Comparison test, *p*<0.05). As the cumulative curve shows, 16.66% of animals exposed to 10 mM PTZ and 41.66% of animals exposed to DZP/10 mM, did not present score 3 prior to the first score 4. The time required to reach score 1–5 is shown in the Figure 2B. Score 5 was observed in 58.33% of the animals pretreated with DZP, and exhibited lower seizure intensity during the entire observation (Student’s *t* test, *p*<0.0001), (Figure 2C and S3). The DZP/10 mM PTZ group presented longer latency than the 10 mM PTZ group to reach the score 4 (Student’s *t* test, *p*<0.0001), (Figure 2D). Concerning the washout period (Figure 3), the time to return to score 0 was similar for animals treated with DZP/10 mM PTZ and 10 mM PTZ.

**Figure 5:**
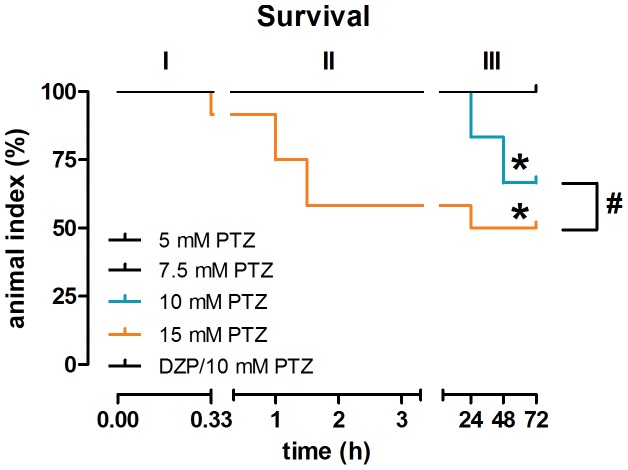
Survival evaluation. Kaplan–Meier plot representing the animal index (%) that survived in 3 distinct periods: I – PTZ exposure; II – Washout period; III – survival evaluation in each 24 h after behavioral experiment procedures. Data are analyzed using the log-rank test for trend to compare groups. *Indicate significant differences from 100% survival, whereas the symbol (#) represents statistical difference profile between 10 and 15 mM PTZ groups.

Only animals under the highest concentration (15 mM) died (score 6) during exposure time and washout period. In the entire experiment, mortality rate was 33.33% and 50% at 10 mM and 15 mM, respectively. Log-rank test for trend indicates a different survival profile between these treatments (X^2^ [1] = 16.20, *p*<0.0001), and Hazard Ratio assumes that the mortality rate occurred 2.25 faster at 15 mM than at 10 mM PTZ. All other treatments, including the DZP pretreatment, had 100% of survival (Figure 5). After 72 h, survival was stable for all treatments (data not shown).

## Discussion

In the current study, we described a detailed behavioral characterization of the adult zebrafish epileptic seizure model induced by PTZ. In the past decade, Baraban et al. [20] demonstrated that PTZ elicits seizures in zebrafish larvae. More recently, several studies have extended this model to adult zebrafish, using three main analyses: electrophysiological evaluation in immobilized animals [39], *c-fos* expression in the CNS [37], and behavioral endpoint parameters [22]. However, a detailed description of the behavioral sequence profile of PTZ-induced seizures in free-swimming zebrafish has not been performed. To our knowledge, this is the first study to describe a temporal behavioral characterization of PTZ-induced seizures and to measure PTZ levels in the brain of adult zebrafish. Both parameters may contribute to make this model useful not only for new AED discovery, but also to elucidate the molecular, neurochemical, and cellular mechanisms underlying seizures.

Among the different methodological approaches described in the literature to define seizure stage-scores, we decided to use a wide range of PTZ concentrations, similarly to the method purposed for larvae zebrafish PTZ model [20] and adult zebrafish KA model [21]. This approach of using different concentrations of PTZ was probably the main reason why distinct scores of seizures were different from what was previously suggested by Desmond et al. [35]. In his study, the authors presented a score system based mostly on one concentration of pro-convulsant drugs exposure (11 mM PTZ, 250 mg/L caffeine, and 0.17 mM picrotoxin). Likewise, we found that exposing the zebrafish to high PTZ concentration, such as 10 mM, our seizure score 3 was skipped, jumping directly from score 2 to 4, in some animals. Similar results can be verified in rodent models when they are injected with high doses of PTZ [40]. Moreover, Desmond et al. [35] define the initial score as freezing and hyperventilation (score 1). At any point the authors described circular behavior and/or whirlpool-like swimming as an independent manifestation from clonic-like seizure. On the other hand, like Baraban et al. (2005), we defined the initial increased activity with hyperventilation as score 1. To better clarify each seizure stage, we divided the scores 3 and 4 from Desmond et al. [35] (circular and/or spiral swimming, rapid movements from left to right, erratic movements, abnormal spasm-like muscular contractions, rapid whole-body clonic-like convulsions and tonic seizures with rigid extension of the body, loss of body posture, sinking to the bottom of the tank, spasms for several minutes, respectively), into score 3 (circular and/or spiral swimming), score 4 (clonic seizure-like behavior), and score 5 (fall to the bottom of the tank, loss of body posture, and tonic seizure-like behavior) (Table 1). This distinction is crucial since each of these stages were reached in a clearly independent time-period, when zebrafish were exposed to the lowest PTZ concentration tested (5 mM). Additionally, not every animal reached the score 5 with 5 mM and 7.5 mM PTZ, showing that clonic-like seizure behavior occurs independently from tonic-like seizure behavior. Since the score 3 in zebrafish larvae exposed to PTZ [20] is defined as a clonus-like convulsion leading to loss of body posture for 1–3 s, we could not use this score for the adult animal. All these manifestations in larvae occur almost at the same time probably because to its immature CNS. What is define as a immobility in larvae can be interpreted as a rigid and extended body posture (tonic-like seizure) that occur independently from clonus-like convulsion behavior in adult zebrafish. Our data also shows that animals rapidly assume left-to-right and erratic movements in the beginning of the behavioral manifestations (score 2) and prior to the circular movements (score 3). However, it is important to emphasize that the animals return to lower scores during PTZ exposure, even after reaching score 5.

Temporal profiles of seizure behavioral manifestation induced by PTZ present a different sequence of score from KA model [21]. The KA score 3, whirlpool-like swimming, corresponds to our score 1; and the score 5, rapid whole-body clonus-like convulsions, correspond to our score 4. This difference may suggest that it is not possible to apply a single stage-score behavior characterization for distinct chemoconvulsant. Nevertheless, animals that received the highest dose of KA or highest concentration of PTZ evolved directly from score 2 to 4, showing that a temporal behavioral profile occurs in different epileptic seizures models.

Seizure intensity was quantified as the area under curve (Figure 2C) in order to measure how fast the animals reach scores 4 and 5 and remain at these scores. Our results show that this parameter increased with PTZ concentration exposures.

In rodent models, the latency to clonic seizure-like behavior is used in screening studies of new AED discovery [41], [42]. However, our results indicate that the analyses of several parameters, rather than only one, could provide a better and reliable instrument that could be used for screening new drugs. Animals exposed to 7.5 mM PTZ or 10 mM PTZ presented similar latency to reach to score 4, but different seizure intensities (Figures 2D and 2C). Additionally, the seizure score curve and the cumulative curve of animal exposed to 7.5 mM PTZ were variable, but the total seizure intensity was similar to 5 mM and different from 10 mM (Figures 2B and 2A). Therefore, these four above-mentioned parameters (score curve, cumulative curve, intensity and latency) would be the foremost method to interpret behavior in different seizure models.

Despite the described well absorption of PTZ by zebrafish, formal HPLC measurements of PTZ in the brain have never been reported for this model [13]. In our study, PTZ brain levels denote a concentration exposure-dependence, presenting a positive interaction between concentration and time of immersion. Additionally, the PTZ brain level of 3.2 μg/mL (1 mL = 3-brain pool) appears to be closely correlated with the first score 5 plateau (Figure 2A). Importantly, our quantification profile was similar to rodent models where a peak of PTZ concentration in the brain is followed by its decreased to the initial levels without reaching zero concentration after 1 h of exposure [40], [43].

Another aspect to be considered is the recovery time after PTZ exposure. Our focus was not to elucidate a post-ictal period from an electrophysiological point of view, but to characterize only the behavioral phenotype of seizures induced by PTZ. We measured the time to return to score 0 and to remain at this score phenotype for 3 h after to remove from the PTZ solution. Our results showed that animals exposed to 5–10 mM exhibited similar time to return to score 0 and similar brain levels of PTZ at 2.5 min post-exposure and after a 60-min washout, even though they presented a completely different epileptic seizure profile. Rodent models show important neurochemical changes during the ictal and post-ictal periods induced by PTZ. Those changes could be related to a post-ictal depression after seizure clonus-like behavior, a period when the CNS remains refractory to further seizure activity [43], [44], [45]. This could explain why, even with detectable levels of PTZ in the brain, the fish returned to the normal behavior. Further studies should be performed to explain why the behavior returns to score 0 at a similar time for all PTZ concentration, except for 15 mM.

Distinctly from seizure intensity, we defined seizure severity as the sum of seizure intensity, latency to return to score 0, and mortality rate. We observed that the severity of PTZ-induced seizure at 15 mM is higher than 10 mM, despite of similar intensity profiles. The higher severity of seizure induced by 15 mM PTZ is associated with longer latency to return to score 0 and mortality rate of 50%. PTZ at 10 mM induced seizures with an intermediary severity, with mortality rate of 33%. On the other hand, 5 and 7.5 mM PTZ induced seizures with lower severity and no changes in total intensity, latency to return to score 0 or mortality rate. Although 15 mM PTZ appears to be a good concentration to study seizure severity, the increase in mortality rate is an important limiting factor in epileptic seizure models [44]. However, in the work published by Pineda et al. [39], where fish were exposed to 15 mM PTZ for only three minutes, the authors mentioned that the mortality rate was zero. Moreover, they also showed that during a continuous exposure to 15 mM PTZ for 90 min, the neuronal activity increased up to 35 min, and only at 90 min of exposure the recording amplitude reversed approximately to the pattern of flat-line EEG, indicative of brain death.

Besides the article mentioned above, the first publication of adult zebrafish and PTZ used 10 mM PTZ and exposure time of 10 min repeated 3 times (total 30 min) [36]. Following this publication, Wong et al. [22] tested 11 mM PTZ for 20 min; Siebel et al. [38] used distinct concentrations (2.5 mM, 5 mM and 15 mM) during 20 min; and Stewart et al. [37] exposed the fish to 11 mM PTZ solution for 20 min. However, no data regarding mortality rate were addressed by any of these publications. Here we show that the amount of PTZ brain levels depend on the exposure time to this drug. The increase in severity for 15 mM PTZ may be attributed to the longer exposure time-period. So, we decided to use 20 min of PTZ exposure and the range of 5, 7.5, 10 and 15 mM PTZ based mostly on these previous literature, aiming to perform a detailed seizure behavior characterization for future studies of this model. As our data showed similar low severity for 5 and 7.5 mM, smaller concentrations (2.5 and 3.75 mM) were not used. This approach of using 20 min and a low concentration of exposure, could be interesting to neurochemical mapping the seizure, since the dopaminergic and serotoninergic [46], cholinergic [47], purinergic [48], and glutamatergic [16] systems have been already described in adult zebrafish.

Due to the great variability of the seizure induced by 7.5 mM PTZ, confirmed by the scatter plot (Figure S2) and the mortality rate of 15 mM, we chose the concentrations of 5 and 10 mM to quantify the levels of PTZ in the brain. Since the mortality rate of 10 mM PTZ induced-seizure were similar to 6 mg/kg KA injection [14] and lower than 15 mM PTZ in adult zebrafish, we decided to use 10 mM PTZ concentration to test all parameters of seizure severity, such as, score curve profile, and latency to reach score 4. These parameters were also tested, when fish are pretreated with a classical anticonvulsant, DZP, to test this behavioral tools for future use in AED research. We observed a decrease in seizure severity in the pretreatment with DZP when compared with 10 mM PTZ treatment alone. The difference in seizure severity for pretreatment with DZP and for all PTZ exposure concentrations could be mathematically demonstrated by the area under the curve analysis, providing a very suitable analysis tool for future AED research. Although the latency to return to score 0 was not affected, it is important to mention that DZP pretreatment presented zero mortality rate.

It is important to point out that the large volume of compounds used in this study (what could be seen as a disadvantage when compared to rodent models), is direct correlated to our main objective. To perform a behavior characterization, a large space is necessary not only for the animals display any behavior alteration, but also to be easier for the observer to detect such alterations. Nevertheless, based on our work, future researchers will be able to have a detailed temporal behavioral repertoire in a smaller volume of PTZ (0.3 L should be enough to perform the same analysis). Considering this small volume or even the intra-peritoneal injection of drugs in adult zebrafish (e.g. MK-801 and DNQX [21]), the amount of AED to be used in future studies may be insignificant when compared to rodent models. Furthermore, the maintenance of zebrafish to investigate the effects of possible anticonvulsant drugs, and mechanisms underlying seizures in future experiments (e.g., behavioral tasks, neurochemical changes, neuronal reorganization and activity) makes zebrafish a lot cheaper and faster than rodent models.

To date, electrophysiology recording in freely swimming adult zebrafish is still a challenge. Despite the studies performed by Pineda et al. [39], [49], the actual EEG methodology is restricted to anesthetized and immobilized animal, making it impossible to correlate the electrophysiological data with the behavior manifestation. We hope that, as soon as new technical approaches emerge to record neuronal activity in freely swimming animal, our behavioral characterization can be used as a background for mapping neuronal activity during the different seizure stages based on the scores proposed by this study.

### Conclusion

In summary, our results described a detailed temporal characterization of the stage-score manifestations of adult zebrafish exposed to distinct PTZ concentrations. We thoroughly described important parameters, such as score curve profile, cumulative score frequency, seizure intensity, latency to score 4 onset, scatter plot score curve, latency to return to score 0, mortality rate and seizure severity. Furthermore, we showed, for the first time, that PTZ brain levels depend on PTZ concentration and exposure time, exhibiting similar profile to rodent models of PTZ injection. Therefore, the behavioral analyses and the simple method for PTZ quantification described here could be considered as important tools for future investigations and translational researches.

## Supporting Information

Figure S1 **PTZ standard curve.** The figure shows the: A) HPLC chromatogram of PTZ detection for different concentrations (1–75 μg/mL); B) linear correlation plot of area for each PTZ concentration.
(TIFF)

Figure S2 **Scatter plot score curve for the experimental groups.** The figure depicts the higher score reached by each animal from 5–15 mM PTZ and DZP/10 mM PTZ groups during the observation time. Each symbol corresponds to its respective animal at each group (*n* = 12). Each symbol represents the profile of a single animal during each interval analyzed and the animal is limited to only one treatment.
(TIFF)

Figure S3 **Comparative score curves.** (A) Overlap of all treatment curves to clarify the 3 moments in the score curves. (B) Seizure intensity for total observation time. Data are represented as mean ± S.E.M and analyzed by one-way ANOVA followed by Bonferroni’s test as post-hoc. Distinct letters indicate statistical difference between PTZ-treated groups (gray bars). The DZP/10 mM PTZ is represented as black bar and compared to 10 mM PTZ group by Student’s *t* test. The asterisks (*) indicates significant difference between both groups.
(TIFF)

Video S1 **Epileptic seizure stage-score induced by PTZ in adult zebrafish.** The video demonstrates the representative behavioral scores (0–6) exhibited by zebrafish exposed to 10 mM PTZ.
(AVI)

## References

[1] Fisher RS, van Emde Boas W, Blume W, Elger C, Genton P, et al (2005) Epileptic seizures and epilepsy: definitions proposed by the International League Against Epilepsy (ILAE) and the International Bureau for Epilepsy (IBE). Epilepsia 46: 470–472. 15816939 10.1111/j.0013-9580.2005.66104.x

[2] Sarkisian MR (2001) Overview of the Current Animal Models for Human Seizure and Epileptic Disorders. Epilepsy Behav 2: 201–216. 12609365 10.1006/ebeh.2001.0193

[3] Racine RJ (1972) Modification of seizure activity by electrical stimulation. II. Motor seizure. Electroencephalogr Clin Neurophysiol 32: 281–294. 4110397 10.1016/0013-4694(72)90177-0

[4] Morimoto K, Fahnestock M, Racine RJ (2004) Kindling and status epilepticus models of epilepsy: rewiring the brain. Prog Neurobiol 73: 1–60. 15193778 10.1016/j.pneurobio.2004.03.009

[5] Loscher W (2011) Critical review of current animal models of seizures and epilepsy used in the discovery and development of new antiepileptic drugs. Seizure 20: 359–368. 21292505 10.1016/j.seizure.2011.01.003

[6] Shorvon SD (2009) Drug treatment of epilepsy in the century of the ILAE: the second 50 years, 1959–2009. Epilepsia 50 Suppl 393–130. 19298435 10.1111/j.1528-1167.2009.02042.x

[7] Shorvon SD (2009) Drug treatment of epilepsy in the century of the ILAE: the first 50 years, 1909–1958. Epilepsia 50 Suppl 369–92. 10.1111/j.1528-1167.2009.02041.x 19298434

[8] Loscher W, Schmidt D (2011) Modern antiepileptic drug development has failed to deliver: ways out of the current dilemma. Epilepsia 52: 657–678. 21426333 10.1111/j.1528-1167.2011.03024.x

[9] Kwan P, Arzimanoglou A, Berg AT, Brodie MJ, Allen Hauser W, et al (2010) Definition of drug resistant epilepsy: consensus proposal by the ad hoc Task Force of the ILAE Commission on Therapeutic Strategies. Epilepsia 51: 1069–1077. 19889013 10.1111/j.1528-1167.2009.02397.x

[10] Penberthy WT, Shafizadeh E, Lin S (2002) The zebrafish as a model for human disease. Front Biosci 7: d1439–1453. 12045008 10.2741/penber

[11] Kari G, Rodeck U, Dicker AP (2007) Zebrafish: an emerging model system for human disease and drug discovery. Clin Pharmacol Ther 82: 70–80. 17495877 10.1038/sj.clpt.6100223

[12] Ingham PW (2009) The power of the zebrafish for disease analysis. Hum Mol Genet 18: R107–112. 19297397 10.1093/hmg/ddp091

[13] Berghmans S, Hunt J, Roach A, Goldsmith P (2007) Zebrafish offer the potential for a primary screen to identify a wide variety of potential anticonvulsants. Epilepsy Res 75: 18–28. 17485198 10.1016/j.eplepsyres.2007.03.015

[14] Sierra S, Alfaro JM, Sanchez S, Burgos JS (2012) Administration of docosahexaenoic acid before birth and until aging decreases kainate-induced seizures in adult zebrafish. Brain Res Bull 88: 467–470. 22542883 10.1016/j.brainresbull.2012.04.007

[15] Barbazuk WB, Korf I, Kadavi C, Heyen J, Tate S, et al (2000) The syntenic relationship of the zebrafish and human genomes. Genome Res 10: 1351–1358. 10984453 10.1101/gr.144700 PMC310919

[16] Rico EP, de Oliveira DL, Rosemberg DB, Mussulini BH, Bonan CD, et al (2010) Expression and functional analysis of Na(+)-dependent glutamate transporters from zebrafish brain. Brain Res Bull 81: 517–523. 19941938 10.1016/j.brainresbull.2009.11.011

[17] Eliceiri BP, Gonzalez AM, Baird A (2011) Zebrafish model of the blood-brain barrier: morphological and permeability studies. Methods Mol Biol 686: 371–378. 21082382 10.1007/978-1-60761-938-3_18 PMC4222041

[18] Jeong JY, Kwon HB, Ahn JC, Kang D, Kwon SH, et al (2008) Functional and developmental analysis of the blood-brain barrier in zebrafish. Brain Res Bull 75: 619–628. 18355638 10.1016/j.brainresbull.2007.10.043

[19] Loscher W (1984) Genetic animal models of epilepsy as a unique resource for the evaluation of anticonvulsant drugs. A review. Methods Find Exp Clin Pharmacol 6: 531–547. 6439966

[20] Baraban SC, Taylor MR, Castro PA, Baier H (2005) Pentylenetetrazole induced changes in zebrafish behavior, neural activity and c-fos expression. Neuroscience 131: 759–768. 15730879 10.1016/j.neuroscience.2004.11.031

[21] Alfaro JM, Ripoll-Gomez J, Burgos JS (2011) Kainate administered to adult zebrafish causes seizures similar to those in rodent models. Eur J Neurosci 33: 1252–1255. 21375600 10.1111/j.1460-9568.2011.07622.x

[22] Wong K, Stewart A, Gilder T, Wu N, Frank K, et al (2010) Modeling seizure-related behavioral and endocrine phenotypes in adult zebrafish. Brain Res 1348: 209–215. 20547142 10.1016/j.brainres.2010.06.012

[23] Hortopan GA, Dinday MT, Baraban SC (2010) Spontaneous seizures and altered gene expression in GABA signaling pathways in a mind bomb mutant zebrafish. J Neurosci 30: 13718–13728. 20943912 10.1523/JNEUROSCI.1887-10.2010 PMC2962868

[24] Hortopan GA, Dinday MT, Baraban SC (2010) Zebrafish as a model for studying genetic aspects of epilepsy. Dis Model Mech 3: 144–148. 20212082 10.1242/dmm.002139

[25] Teng Y, Xie X, Walker S, Saxena M, Kozlowski DJ, et al (2011) Loss of zebrafish lgi1b leads to hydrocephalus and sensitization to pentylenetetrazol induced seizure-like behavior. PLoS One 6: e24596. 22053218 10.1371/journal.pone.0024596 PMC3203530

[26] Baraban SC, Dinday MT, Castro PA, Chege S, Guyenet S, et al (2007) A large-scale mutagenesis screen to identify seizure-resistant zebrafish. Epilepsia 48: 1151–1157. 17521353 10.1111/j.1528-1167.2007.01075.x PMC2211740

[27] Bassuk AG, Wallace RH, Buhr A, Buller AR, Afawi Z, et al (2008) A homozygous mutation in human PRICKLE1 causes an autosomal-recessive progressive myoclonus epilepsy-ataxia syndrome. Am J Hum Genet 83: 572–581. 18976727 10.1016/j.ajhg.2008.10.003 PMC2668041

[28] DiBella LM, Park A, Sun Z (2009) Zebrafish Tsc1 reveals functional interactions between the cilium and the TOR pathway. Hum Mol Genet 18: 595–606. 19008302 10.1093/hmg/ddn384 PMC2722215

[29] Teng Y, Xie X, Walker S, Rempala G, Kozlowski DJ, et al (2010) Knockdown of zebrafish Lgi1a results in abnormal development, brain defects and a seizure-like behavioral phenotype. Hum Mol Genet 19: 4409–4420. 20819949 10.1093/hmg/ddq364 PMC2957326

[30] Rosemberg DB, Rico EP, Mussulini BH, Piato AL, Calcagnotto ME, et al (2011) Differences in spatio-temporal behavior of zebrafish in the open tank paradigm after a short-period confinement into dark and bright environments. PLoS One 6: e19397. 21559304 10.1371/journal.pone.0019397 PMC3085514

[31] Blaser RE, Rosemberg DB (2012) Measures of anxiety in zebrafish (Danio rerio): dissociation of black/white preference and novel tank test. PLoS One 7: e36931. 22615849 10.1371/journal.pone.0036931 PMC3355173

[32] Rosemberg DB, Braga MM, Rico EP, Loss CM, Cordova SD, et al (2012) Behavioral effects of taurine pretreatment in zebrafish acutely exposed to ethanol. Neuropharmacology 63: 613–623. 22634362 10.1016/j.neuropharm.2012.05.009

[33] Cachat J, Stewart A, Utterback E, Hart P, Gaikwad S, et al (2011) Three-dimensional neurophenotyping of adult zebrafish behavior. PLoS One 6: e17597. 21408171 10.1371/journal.pone.0017597 PMC3049776

[34] Muller UK, van Leeuwen JL (2004) Swimming of larval zebrafish: ontogeny of body waves and implications for locomotory development. J Exp Biol 207: 853–868. 14747416 10.1242/jeb.00821

[35] Desmond D, Kyzar E, Gaikwad S, Green J, Riehl R, et al.. (2012) Assessing epilepsy-related behavioral phenotypes in adult zebrafish.; Press H, editor. New York.

[36] Lee Y, Kim D, Kim YH, Lee H, Lee CJ (2010) Improvement of pentylenetetrazol-induced learning deficits by valproic acid in the adult zebrafish. Eur J Pharmacol 643: 225–231. 20599908 10.1016/j.ejphar.2010.06.041

[37] Stewart AM, Desmond D, Kyzar E, Gaikwad S, Roth A, et al (2012) Perspectives of zebrafish models of epilepsy: what, how and where next? Brain Res Bull 87: 135–143. 22155548 10.1016/j.brainresbull.2011.11.020

[38] Siebel AM, Piato AL, Capiotti KM, Seibt KJ, Bogo MR, et al (2011) PTZ-induced seizures inhibit adenosine deamination in adult zebrafish brain membranes. Brain Res Bull 86: 385–389. 21907764 10.1016/j.brainresbull.2011.08.017

[39] Pineda R, Beattie CE, Hall CW (2011) Recording the adult zebrafish cerebral field potential during pentylenetetrazole seizures. J Neurosci Methods 200: 20–28. 21689682 10.1016/j.jneumeth.2011.06.001 PMC5503205

[40] Soto-Otero R, Mendez-Alvarez E, Sierra-Paredes G, Galan-Valiente J, Aguilar-Veiga E, et al (1987) High-performance liquid chromatographic procedure for quantitative determination of pentylenetetrazol in serum and discrete areas of rat brain. Anal Biochem 165: 331–336. 3425901 10.1016/0003-2697(87)90277-6

[41] Vermoesen K, Serruys AS, Loyens E, Afrikanova T, Massie A, et al (2011) Assessment of the convulsant liability of antidepressants using zebrafish and mouse seizure models. Epilepsy Behav 22: 450–460. 21962757 10.1016/j.yebeh.2011.08.016

[42] Bachiega JC, Blanco MM, Perez-Mendes P, Cinini SM, Covolan L, et al (2008) Behavioral characterization of pentylenetetrazol-induced seizures in the marmoset. Epilepsy Behav 13: 70–76. 18337181 10.1016/j.yebeh.2008.02.010

[43] Ramzan IM, Levy G (1985) Kinetics of drug action in disease states. XIV. Effect of infusion rate on pentylenetetrazol concentrations in serum, brain and cerebrospinal fluid of rats at onset of convulsions. J Pharmacol Exp Ther 234: 624–628. 3875710

[44] Goodman LS, Grewal MS, Brown WC, Swinyard EA (1953) Comparison of maximal seizures evoked by pentylenetetrazol (metrazol) and electroshock in mice, and their modification by anticonvulsants. J Pharmacol Exp Ther 108: 168–176. 13062087

[45] Yonekawa WD, Kupferberg HJ, Woodbury DM (1980) Relationship between pentylenetetrazol-induced seizures and brain pentylenetetrazol levels in mice. J Pharmacol Exp Ther 214: 589–593. 7400961

[46] Chatterjee D, Gerlai R (2009) High precision liquid chromatography analysis of dopaminergic and serotoninergic responses to acute alcohol exposure in zebrafish. Behav Brain Res 200: 208–213. 19378384 10.1016/j.bbr.2009.01.016 PMC2709823

[47] Rico EP, Rosemberg DB, Dias RD, Bogo MR, Bonan CD (2007) Ethanol alters acetylcholinesterase activity and gene expression in zebrafish brain. Toxicol Lett 174: 25–30. 17888594 10.1016/j.toxlet.2007.08.005

[48] Rosemberg DB, Rico EP, Senger MR, Dias RD, Bogo MR, et al (2008) Kinetic characterization of adenosine deaminase activity in zebrafish (Danio rerio) brain. Comp Biochem Physiol B Biochem Mol Biol 151: 96–101. 18582589 10.1016/j.cbpb.2008.06.001

[49] Pineda R, Beattie CE, Hall CW (2012) Closed-loop neural stimulation for pentylenetetrazole-induced seizures in zebrafish. Dis Model Mech. 10.1242/dmm.009423 PMC3529339 22822044

