# Supplementary figures and images for: Seizures Induced by Pentylenetetrazole in the Adult Zebrafish: A Detailed Behavioral Characterization
Source: PLoS One. 2013 Jan 21;8(1):e54515. doi: 10.1371/journal.pone.0054515 (PMC3549980; doi:10.1371/journal.pone.0054515)

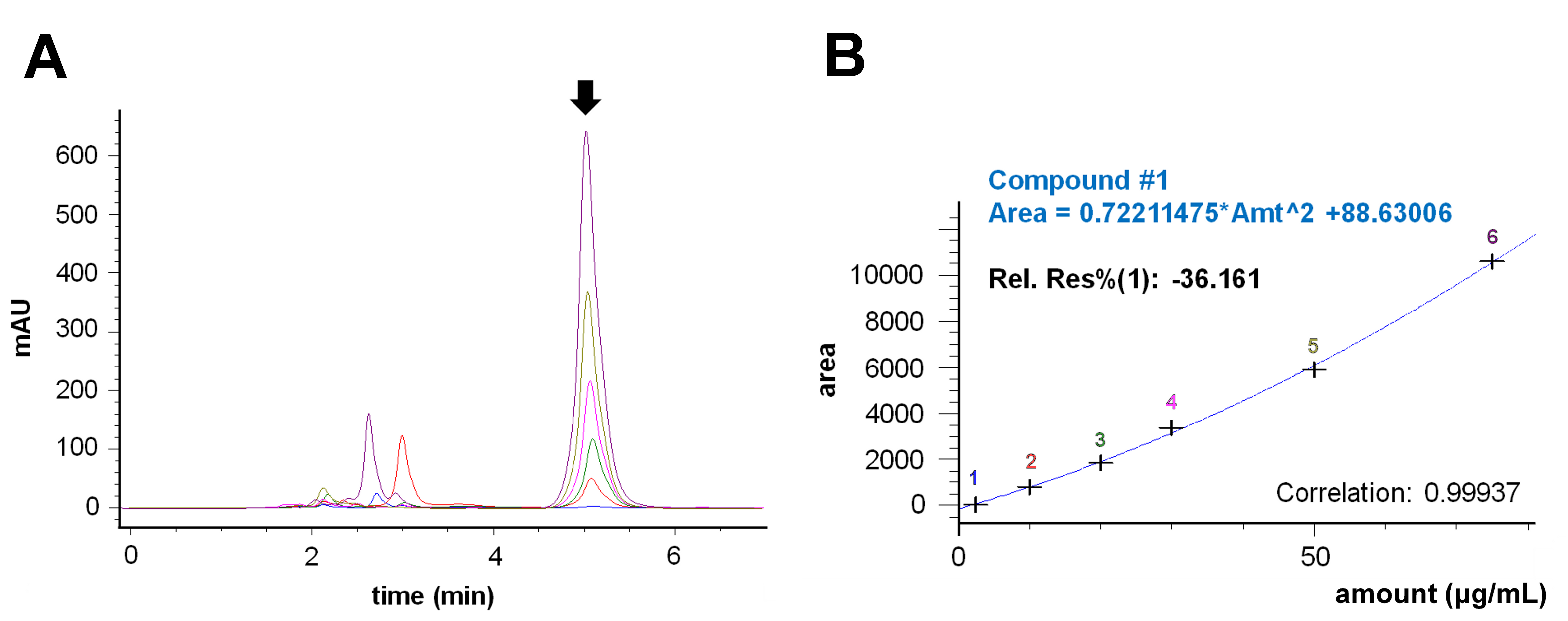

Supplement: Figure S1 — PTZ standard curve. The figure shows the: A) HPLC chromatogram of PTZ detection for different concentrations (1–75 μg/mL); B) linear correlation plot of area for each PTZ concentration. (TIFF) [file pone.0054515.s001.tif]

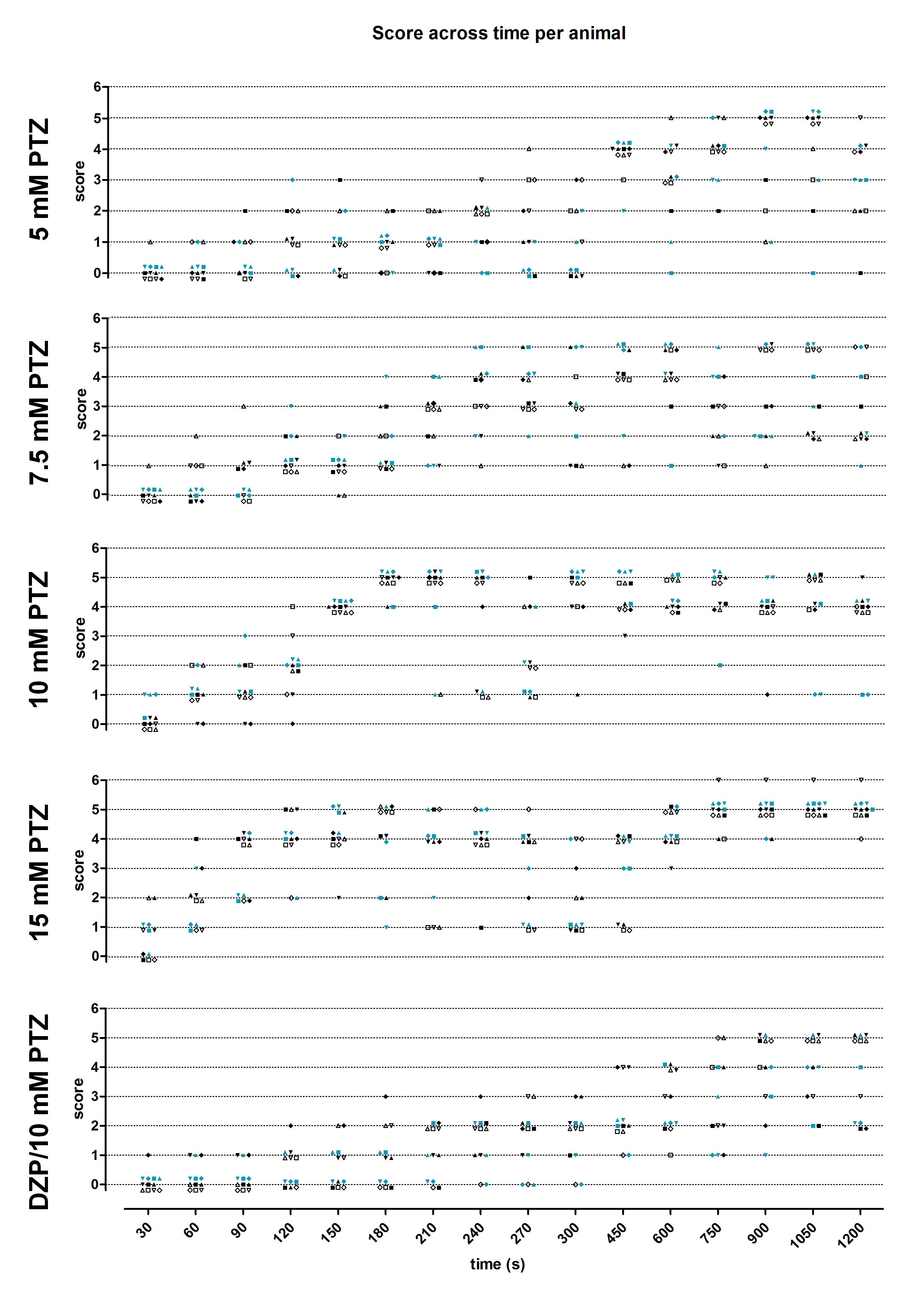

Supplement: Figure S2 — Scatter plot score curve for the experimental groups. The figure depicts the higher score reached by each animal from 5–15 mM PTZ and DZP/10 mM PTZ groups during the observation time. Each symbol corresponds to its respective animal at each group (n = 12). Each symbol represents the profile of a single animal during each interval analyzed and the animal is limited to only one treatment. (TIFF) [file pone.0054515.s002.tif]

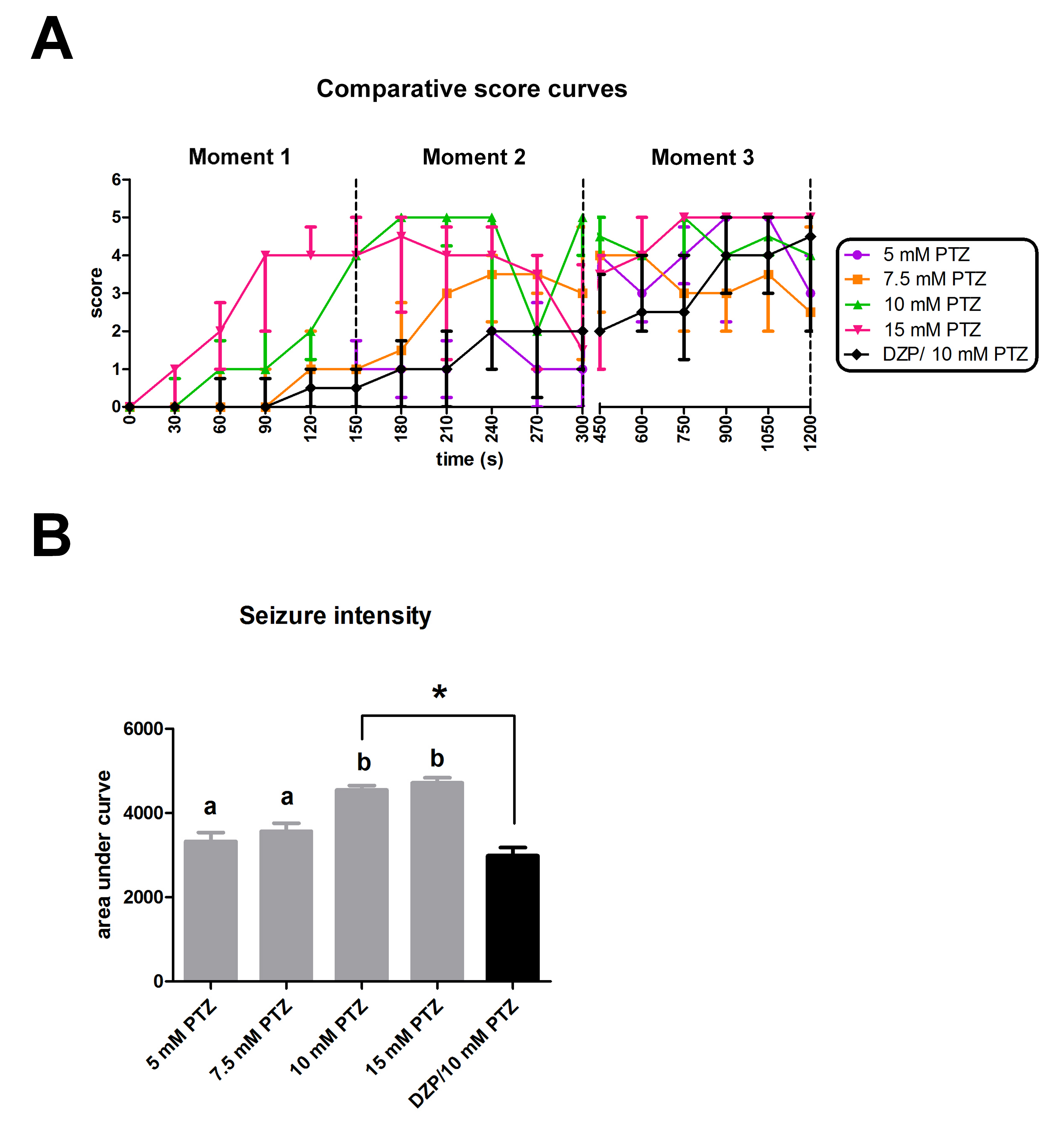

Supplement: Figure S3 — Comparative score curves. (A) Overlap of all treatment curves to clarify the 3 moments in the score curves. (B) Seizure intensity for total observation time. Data are represented as mean ± S.E.M and analyzed by one-way ANOVA followed by Bonferroni’s test as post-hoc. Distinct letters indicate statistical difference between PTZ-treated groups (gray bars). The DZP/10 mM PTZ is represented as black bar and compared to 10 mM PTZ group by Student’s t test. The asterisks (*) indicates significant difference between both groups. (TIFF) [file pone.0054515.s003.tif]
